# Tricyclo­hexyl(piperidine-1-dithio­carboxyl­ato-κ*S*)tin(IV)

**DOI:** 10.1107/S1600536808021909

**Published:** 2008-07-16

**Authors:** Lujiang Hao, Chunhua Mu, Binbin Kong

**Affiliations:** a College of Food and Biological Engineering, Shandong Institute of Light Industry, Jinan 250353, People’s Republic of China; b Maize Research Insitute, Shandong Academy of Agricultural Science, Jinan 250100, People’s Republic of China

## Abstract

In the title compound, [Sn(C_6_H_11_)_3_(C_6_H_10_NS_2_)], the Sn^IV^ atom is tetra­coordinated by three C atoms from cyclo­hexyl groups and one S atom from a piperidine­dithio­carboxyl­ate anion. The coordination geometry is distorted tetra­hedral, with Sn—C bond lengths in the range 2.133 (6)–2.188 (6) Å and with an Sn—S bond length of 2.4516 (19) Å. The nonbonded S atom of the piperidine­dithio­carboxyl­ate anion makes an Sn⋯S contact of 3.174 (3) Å.

## Related literature

For related literature, see: Church & Halvorson (1959 ▶); Chung *et al.* (1971 ▶); Okabe & Oya (2000 ▶); Serre *et al.* (2005 ▶); Pocker & Fong (1980 ▶); Scapin *et al.* (1997 ▶).
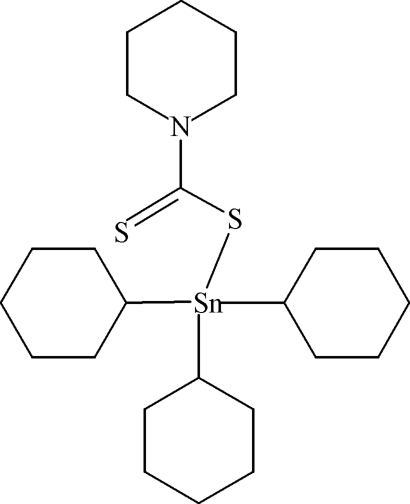


## Experimental

### 

#### Crystal data

[Sn(C_6_H_11_)_3_(C_6_H_10_NS_2_)]
*M* *_r_* = 528.40
Monoclinic, 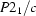

*a* = 17.227 (8) Å
*b* = 7.676 (4) Å
*c* = 20.827 (10) Å
β = 109.598 (8)°
*V* = 2594 (2) Å^3^
*Z* = 4
Mo *K*α radiation
μ = 1.16 mm^−1^
*T* = 295 (2) K
0.26 × 0.20 × 0.12 mm

#### Data collection

Bruker APEXII CCD diffractometer
Absorption correction: multi-scan (*SADABS*; Bruker, 2001 ▶) *T*_min_ = 0.753, *T*_max_ = 0.874
22395 measured reflections
4846 independent reflections
3085 reflections with *I* > 2σ(*I*)
*R*_int_ = 0.081

#### Refinement

*R*[*F* ^2^ > 2σ(*F* ^2^)] = 0.053
*wR*(*F* ^2^) = 0.141
*S* = 1.04
4846 reflections
253 parameters
36 restraints
H-atom parameters constrained
Δρ_max_ = 0.76 e Å^−3^
Δρ_min_ = −0.66 e Å^−3^

### 

Data collection: *APEX2* (Bruker, 2004 ▶); cell refinement: *SAINT-Plus* (Bruker, 2001 ▶); data reduction: *SAINT-Plus*; program(s) used to solve structure: *SHELXS97* (Sheldrick, 2008 ▶); program(s) used to refine structure: *SHELXL97* (Sheldrick, 2008 ▶); molecular graphics: *SHELXTL* (Sheldrick, 2008 ▶); software used to prepare material for publication: *SHELXTL*.

## Supplementary Material

Crystal structure: contains datablocks global, I. DOI: 10.1107/S1600536808021909/bi2291sup1.cif

Structure factors: contains datablocks I. DOI: 10.1107/S1600536808021909/bi2291Isup2.hkl

Additional supplementary materials:  crystallographic information; 3D view; checkCIF report

## Figures and Tables

**Fig. 1.**
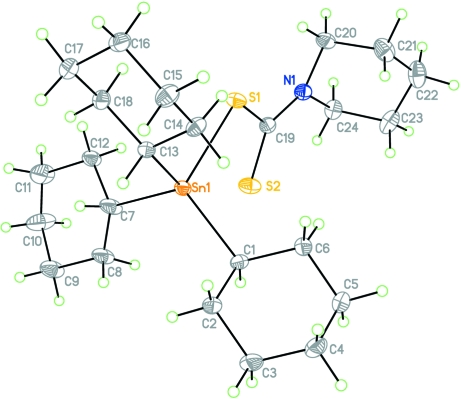
The molecular structure of the title compound, with 30% probability displacement ellipsoids for the non-H atoms.

## supplementary crystallographic information

### Comment

Organotin esters of thiocarboxylic acids are widely used as biocides, fungicides and homogeneous catalysts in industry (Church & Halvorson, 1959; Chung *et al.*, 1971). Recently, pharmaceutical properties of organotin esters of carboxylic acids have been investigated for their anti-tumour activity (Okabe & Oya, 2000; Serre *et al.*, 2005). Studies on organotin compounds containing carboxylate ligands with additional donor atoms (*e.g.* N, O, or S) that are available for coordination to the Sn atom have revealed that new structural types may lead to different activities (Pocker & Fong, 1980; Scapin *et al.*, 1997).

### Experimental

A mixture of tricyclohexyltin bromide (0.5 mmol) and piperidinothiocarboxylic acid (0.5 mmol) in 20 ml methanol solution was refluxed for one hour. The resulting filtrate was evaporated at room temperature for three days. Colourless crystals were obtained with a yield of 21%. Elemental analysis calculated: C 54.50, H 8.14, N 2.65%; found: C 54.44, H 8.12, N 2.58%.

### Refinement

All H atoms were placed in calculated positions with C—H = 0.97 or 0.98 Å and refined as riding with *U*_iso_(H) = 1.2*U*_eq_(C). The 1,2 and 1,3 C—C distances in all cyclohexane rings were restrained to be equivalent with an esd of 0.01 Å.

### Crystal data

**Table:**

[Sn(C_6_H_11_)_3_(C_6_H_10_NS_2_)]	*F*_000_ = 1104
*M**_r_* = 528.40	*D*_x_ = 1.353 Mg m^−^^3^
Monoclinic, *P*2_1_/*c*	Mo *K*α radiation λ = 0.71073 Å
Hall symbol: -P 2ybc	Cell parameters from 4846 reflections
*a* = 17.227 (8) Å	θ = 1.3–25.9º
*b* = 7.676 (4) Å	µ = 1.16 mm^−^^1^
*c* = 20.827 (10) Å	*T* = 295 (2) K
β = 109.598 (8)º	Block, colorless
*V* = 2594 (2) Å^3^	0.26 × 0.20 × 0.12 mm
*Z* = 4	

### Data collection

**Table:**

Bruker APEXII CCD diffractometer	4846 independent reflections
Radiation source: fine-focus sealed tube	3085 reflections with *I* > 2σ(*I*)
Monochromator: graphite	*R*_int_ = 0.081
*T* = 295(2) K	θ_max_ = 25.7º
φ and ω scans	θ_min_ = 1.3º
Absorption correction: multi-scan(SADABS; Bruker, 2001)	*h* = −21→21
*T*_min_ = 0.753, *T*_max_ = 0.874	*k* = −9→9
22395 measured reflections	*l* = −25→25

### Refinement

**Table:**

Refinement on *F*^2^	Secondary atom site location: difference Fourier map
Least-squares matrix: full	Hydrogen site location: inferred from neighbouring sites
*R*[*F*^2^ > 2σ(*F*^2^)] = 0.053	H-atom parameters constrained
*wR*(*F*^2^) = 0.141	*w* = 1/[σ^2^(*F*_o_^2^) + (0.0663*P*)^2^] where *P* = (*F*_o_^2^ + 2*F*_c_^2^)/3
*S* = 1.04	(Δ/σ)_max_ = 0.001
4846 reflections	Δρ_max_ = 0.76 e Å^−^^3^
253 parameters	Δρ_min_ = −0.66 e Å^−^^3^
36 restraints	Extinction correction: none
Primary atom site location: structure-invariant direct methods	

### Special details

**Table:**

Geometry. All esds (except the esd in the dihedral angle between two l.s. planes) are estimated using the full covariance matrix. The cell esds are taken into account individually in the estimation of esds in distances, angles and torsion angles; correlations between esds in cell parameters are only used when they are defined by crystal symmetry. An approximate (isotropic) treatment of cell esds is used for estimating esds involving l.s. planes.
Refinement. Refinement of *F*^2^ against ALL reflections. The weighted *R*-factor *wR* and goodness of fit *S* are based on *F*^2^, conventional *R*-factors *R* are based on *F*, with *F* set to zero for negative *F*^2^. The threshold expression of *F*^2^ > σ(*F*^2^) is used only for calculating *R*-factors(gt) *etc*. and is not relevant to the choice of reflections for refinement. *R*-factors based on *F*^2^ are statistically about twice as large as those based on *F*, and *R*- factors based on ALL data will be even larger.

### Fractional atomic coordinates and isotropic or equivalent isotropic displacement parameters (Å^2^)

**Table:**

	*x*	*y*	*z*	*U*_iso_*/*U*_eq_	
Sn1	0.21447 (3)	0.20684 (5)	0.93872 (2)	0.05978 (19)	
S1	0.29048 (10)	0.4803 (2)	0.97369 (9)	0.0736 (5)	
S2	0.40599 (12)	0.1842 (2)	1.02151 (12)	0.0911 (6)	
N1	0.4422 (3)	0.5166 (7)	1.0553 (3)	0.0670 (13)	
C1	0.2069 (3)	0.0560 (7)	1.0235 (3)	0.0602 (15)	
H1A	0.2511	−0.0307	1.0336	0.072*	
C2	0.1285 (4)	−0.0421 (8)	1.0036 (3)	0.082 (2)	
H2A	0.1246	−0.1152	0.9646	0.098*	
H2B	0.0828	0.0393	0.9900	0.098*	
C3	0.1217 (5)	−0.1548 (9)	1.0610 (4)	0.102 (3)	
H3A	0.0680	−0.2103	1.0470	0.122*	
H3B	0.1632	−0.2456	1.0709	0.122*	
C4	0.1330 (5)	−0.0499 (10)	1.1237 (4)	0.104 (3)	
H4A	0.0876	0.0315	1.1153	0.125*	
H4B	0.1322	−0.1267	1.1604	0.125*	
C5	0.2108 (5)	0.0466 (10)	1.1443 (3)	0.101 (2)	
H5A	0.2562	−0.0355	1.1591	0.121*	
H5B	0.2136	0.1204	1.1829	0.121*	
C6	0.2207 (4)	0.1582 (8)	1.0879 (3)	0.0805 (19)	
H6A	0.1816	0.2537	1.0788	0.097*	
H6B	0.2757	0.2074	1.1025	0.097*	
C7	0.2408 (6)	0.0636 (8)	0.8607 (4)	0.109 (3)	
H7A	0.1857	0.0637	0.8261	0.131*	
C8	0.2528 (7)	−0.1148 (9)	0.8691 (5)	0.161 (5)	
H8A	0.2072	−0.1634	0.8806	0.193*	
H8B	0.3025	−0.1345	0.9077	0.193*	
C9	0.2603 (7)	−0.2145 (9)	0.8092 (5)	0.125 (4)	
H9A	0.2824	−0.3296	0.8239	0.150*	
H9B	0.2062	−0.2284	0.7751	0.150*	
C10	0.3153 (7)	−0.1220 (10)	0.7786 (5)	0.153 (5)	
H10A	0.3715	−0.1293	0.8098	0.184*	
H10B	0.3131	−0.1813	0.7369	0.184*	
C11	0.2952 (7)	0.0542 (11)	0.7636 (4)	0.146 (4)	
H11A	0.2438	0.0607	0.7257	0.175*	
H11B	0.3376	0.1075	0.7490	0.175*	
C12	0.2866 (4)	0.1581 (8)	0.8223 (3)	0.083 (2)	
H12A	0.3410	0.1873	0.8533	0.099*	
H12B	0.2581	0.2662	0.8049	0.099*	
C13	0.0980 (4)	0.3417 (7)	0.8883 (3)	0.0646 (16)	
H13A	0.0558	0.2509	0.8735	0.078*	
C14	0.0716 (4)	0.4589 (8)	0.9331 (3)	0.0723 (18)	
H14A	0.1141	0.5457	0.9522	0.087*	
H14B	0.0654	0.3919	0.9706	0.087*	
C15	−0.0088 (4)	0.5499 (9)	0.8960 (3)	0.089 (2)	
H15A	−0.0529	0.4647	0.8829	0.106*	
H15B	−0.0208	0.6330	0.9265	0.106*	
C16	−0.0060 (4)	0.6428 (8)	0.8339 (3)	0.084 (2)	
H16A	−0.0597	0.6927	0.8100	0.101*	
H16B	0.0335	0.7374	0.8473	0.101*	
C17	0.0175 (4)	0.5240 (9)	0.7877 (3)	0.090 (2)	
H17A	0.0230	0.5897	0.7497	0.108*	
H17B	−0.0254	0.4377	0.7697	0.108*	
C18	0.0983 (4)	0.4331 (8)	0.8250 (3)	0.0762 (18)	
H18A	0.1097	0.3489	0.7946	0.091*	
H18B	0.1423	0.5185	0.8370	0.091*	
C19	0.3871 (4)	0.3993 (8)	1.0215 (3)	0.0638 (15)	
C20	0.4243 (4)	0.6994 (8)	1.0636 (4)	0.079 (2)	
H20A	0.3724	0.7314	1.0293	0.095*	
H20B	0.4673	0.7721	1.0573	0.095*	
C21	0.4195 (6)	0.7289 (11)	1.1325 (5)	0.112 (3)	
H21A	0.3723	0.6671	1.1368	0.135*	
H21B	0.4120	0.8522	1.1389	0.135*	
C22	0.4978 (6)	0.6655 (15)	1.1871 (5)	0.132 (4)	
H22A	0.5428	0.7433	1.1887	0.158*	
H22B	0.4901	0.6700	1.2311	0.158*	
C23	0.5204 (5)	0.4844 (13)	1.1745 (4)	0.112 (3)	
H23A	0.4805	0.4035	1.1812	0.135*	
H23B	0.5740	0.4559	1.2071	0.135*	
C24	0.5228 (4)	0.4648 (10)	1.1035 (3)	0.082 (2)	
H24A	0.5658	0.5379	1.0975	0.098*	
H24B	0.5345	0.3447	1.0954	0.098*	

### Atomic displacement parameters (Å^2^)

**Table:**

	*U*^11^	*U*^22^	*U*^33^	*U*^12^	*U*^13^	*U*^23^
Sn1	0.0764 (3)	0.0486 (3)	0.0632 (3)	−0.0086 (2)	0.0351 (2)	−0.0032 (2)
S1	0.0691 (10)	0.0512 (9)	0.0971 (13)	−0.0085 (8)	0.0233 (9)	0.0023 (9)
S2	0.0931 (13)	0.0608 (11)	0.1322 (18)	0.0064 (9)	0.0547 (13)	0.0058 (11)
N1	0.063 (3)	0.067 (3)	0.075 (4)	−0.007 (3)	0.029 (3)	0.002 (3)
C1	0.070 (4)	0.049 (3)	0.069 (4)	0.004 (3)	0.034 (3)	0.008 (3)
C2	0.092 (5)	0.077 (5)	0.078 (5)	−0.027 (4)	0.030 (4)	0.005 (4)
C3	0.122 (6)	0.087 (5)	0.112 (7)	−0.029 (5)	0.060 (5)	0.010 (5)
C4	0.135 (7)	0.109 (6)	0.089 (6)	−0.007 (6)	0.065 (5)	0.015 (5)
C5	0.137 (7)	0.102 (6)	0.066 (5)	−0.016 (5)	0.039 (5)	0.000 (4)
C6	0.102 (5)	0.073 (4)	0.070 (4)	−0.018 (4)	0.034 (4)	−0.007 (4)
C7	0.193 (9)	0.063 (4)	0.121 (7)	−0.025 (5)	0.119 (7)	−0.027 (4)
C8	0.311 (15)	0.068 (5)	0.196 (11)	−0.029 (7)	0.206 (11)	−0.026 (6)
C9	0.203 (10)	0.075 (5)	0.147 (9)	−0.026 (6)	0.123 (8)	−0.029 (5)
C10	0.274 (13)	0.083 (6)	0.185 (10)	0.034 (8)	0.187 (11)	0.022 (7)
C11	0.242 (12)	0.128 (8)	0.126 (8)	−0.010 (8)	0.138 (9)	−0.002 (7)
C12	0.112 (6)	0.064 (4)	0.090 (5)	−0.017 (4)	0.059 (5)	−0.008 (4)
C13	0.072 (4)	0.059 (4)	0.062 (4)	−0.015 (3)	0.021 (3)	0.000 (3)
C14	0.076 (4)	0.083 (5)	0.067 (4)	0.007 (4)	0.036 (4)	0.017 (4)
C15	0.089 (5)	0.104 (6)	0.084 (5)	0.010 (4)	0.044 (4)	0.017 (4)
C16	0.105 (5)	0.069 (4)	0.080 (5)	0.008 (4)	0.035 (4)	0.013 (4)
C17	0.111 (6)	0.084 (5)	0.066 (5)	0.005 (4)	0.019 (4)	0.007 (4)
C18	0.098 (5)	0.074 (4)	0.063 (4)	0.000 (4)	0.035 (4)	−0.002 (3)
C19	0.063 (4)	0.069 (4)	0.068 (4)	−0.008 (3)	0.034 (3)	0.002 (3)
C20	0.078 (4)	0.068 (4)	0.099 (6)	−0.015 (4)	0.038 (4)	−0.004 (4)
C21	0.102 (7)	0.117 (7)	0.135 (8)	−0.005 (5)	0.062 (6)	−0.034 (6)
C22	0.116 (8)	0.190 (11)	0.099 (7)	−0.008 (8)	0.048 (6)	−0.039 (7)
C23	0.093 (6)	0.162 (9)	0.085 (6)	0.015 (6)	0.034 (5)	0.025 (6)
C24	0.060 (4)	0.105 (6)	0.082 (5)	−0.002 (4)	0.026 (4)	−0.002 (4)

### Geometric parameters (Å, °)

**Table:**

Sn1—C7	2.133 (6)	C10—H10B	0.970
Sn1—C1	2.151 (5)	C11—C12	1.509 (7)
Sn1—C13	2.188 (6)	C11—H11A	0.970
Sn1—S1	2.4516 (19)	C11—H11B	0.970
Sn1—S2	3.174 (3)	C12—H12A	0.970
S1—C19	1.742 (6)	C12—H12B	0.970
S2—C19	1.683 (7)	C13—C14	1.475 (6)
N1—C19	1.327 (7)	C13—C18	1.493 (6)
N1—C20	1.459 (8)	C13—H13A	0.980
N1—C24	1.469 (8)	C14—C15	1.512 (7)
C1—C2	1.480 (7)	C14—H14A	0.970
C1—C6	1.502 (7)	C14—H14B	0.970
C1—H1A	0.980	C15—C16	1.492 (7)
C2—C3	1.512 (7)	C15—H15A	0.970
C2—H2A	0.970	C15—H15B	0.970
C2—H2B	0.970	C16—C17	1.477 (7)
C3—C4	1.490 (8)	C16—H16A	0.970
C3—H3A	0.970	C16—H16B	0.970
C3—H3B	0.970	C17—C18	1.517 (7)
C4—C5	1.463 (7)	C17—H17A	0.970
C4—H4A	0.970	C17—H17B	0.970
C4—H4B	0.970	C18—H18A	0.970
C5—C6	1.510 (7)	C18—H18B	0.970
C5—H5A	0.970	C20—C21	1.484 (11)
C5—H5B	0.970	C20—H20A	0.970
C6—H6A	0.970	C20—H20B	0.970
C6—H6B	0.970	C21—C22	1.523 (12)
C7—C8	1.387 (7)	C21—H21A	0.970
C7—C12	1.487 (6)	C21—H21B	0.970
C7—H7A	0.980	C22—C23	1.490 (11)
C8—C9	1.506 (8)	C22—H22A	0.970
C8—H8A	0.970	C22—H22B	0.970
C8—H8B	0.970	C23—C24	1.501 (10)
C9—C10	1.487 (8)	C23—H23A	0.970
C9—H9A	0.970	C23—H23B	0.970
C9—H9B	0.970	C24—H24A	0.970
C10—C11	1.405 (8)	C24—H24B	0.970
C10—H10A	0.970		
			
C7—Sn1—C1	115.8 (2)	C10—C11—H11B	108.7
C7—Sn1—C13	105.8 (3)	C12—C11—H11B	108.7
C1—Sn1—C13	110.5 (2)	H11A—C11—H11B	107.6
C7—Sn1—S1	116.04 (19)	C7—C12—C11	113.1 (5)
C1—Sn1—S1	112.96 (15)	C7—C12—H12A	109.0
C13—Sn1—S1	92.86 (16)	C11—C12—H12A	109.0
C7—Sn1—S2	86.4 (3)	C7—C12—H12B	109.0
C1—Sn1—S2	82.11 (15)	C11—C12—H12B	109.0
C13—Sn1—S2	154.88 (16)	H12A—C12—H12B	107.8
S1—Sn1—S2	62.03 (5)	C14—C13—C18	111.4 (5)
C19—S1—Sn1	100.2 (2)	C14—C13—Sn1	114.4 (4)
C19—S2—Sn1	77.3 (2)	C18—C13—Sn1	111.9 (4)
C19—N1—C20	124.9 (6)	C14—C13—H13A	106.2
C19—N1—C24	121.6 (6)	C18—C13—H13A	106.2
C20—N1—C24	111.6 (6)	Sn1—C13—H13A	106.2
C2—C1—C6	111.8 (5)	C13—C14—C15	112.5 (5)
C2—C1—Sn1	110.2 (4)	C13—C14—H14A	109.1
C6—C1—Sn1	114.6 (4)	C15—C14—H14A	109.1
C2—C1—H1A	106.6	C13—C14—H14B	109.1
C6—C1—H1A	106.6	C15—C14—H14B	109.1
Sn1—C1—H1A	106.6	H14A—C14—H14B	107.8
C1—C2—C3	112.0 (5)	C16—C15—C14	111.9 (5)
C1—C2—H2A	109.2	C16—C15—H15A	109.2
C3—C2—H2A	109.2	C14—C15—H15A	109.2
C1—C2—H2B	109.2	C16—C15—H15B	109.2
C3—C2—H2B	109.2	C14—C15—H15B	109.2
H2A—C2—H2B	107.9	H15A—C15—H15B	107.9
C4—C3—C2	111.2 (6)	C17—C16—C15	111.4 (5)
C4—C3—H3A	109.4	C17—C16—H16A	109.3
C2—C3—H3A	109.4	C15—C16—H16A	109.3
C4—C3—H3B	109.4	C17—C16—H16B	109.3
C2—C3—H3B	109.4	C15—C16—H16B	109.3
H3A—C3—H3B	108.0	H16A—C16—H16B	108.0
C5—C4—C3	111.6 (6)	C16—C17—C18	110.8 (5)
C5—C4—H4A	109.3	C16—C17—H17A	109.5
C3—C4—H4A	109.3	C18—C17—H17A	109.5
C5—C4—H4B	109.3	C16—C17—H17B	109.5
C3—C4—H4B	109.3	C18—C17—H17B	109.5
H4A—C4—H4B	108.0	H17A—C17—H17B	108.1
C4—C5—C6	112.9 (6)	C13—C18—C17	113.3 (5)
C4—C5—H5A	109.0	C13—C18—H18A	108.9
C6—C5—H5A	109.0	C17—C18—H18A	108.9
C4—C5—H5B	109.0	C13—C18—H18B	108.9
C6—C5—H5B	109.0	C17—C18—H18B	108.9
H5A—C5—H5B	107.8	H18A—C18—H18B	107.7
C1—C6—C5	112.0 (5)	N1—C19—S2	124.2 (5)
C1—C6—H6A	109.2	N1—C19—S1	116.0 (5)
C5—C6—H6A	109.2	S2—C19—S1	119.8 (4)
C1—C6—H6B	109.2	N1—C20—C21	110.1 (6)
C5—C6—H6B	109.2	N1—C20—H20A	109.6
H6A—C6—H6B	107.9	C21—C20—H20A	109.6
C8—C7—C12	117.7 (6)	N1—C20—H20B	109.6
C8—C7—Sn1	118.3 (5)	C21—C20—H20B	109.6
C12—C7—Sn1	116.4 (4)	H20A—C20—H20B	108.2
C8—C7—H7A	99.2	C20—C21—C22	110.5 (7)
C12—C7—H7A	99.2	C20—C21—H21A	109.5
Sn1—C7—H7A	99.2	C22—C21—H21A	109.5
C7—C8—C9	116.6 (7)	C20—C21—H21B	109.5
C7—C8—H8A	108.1	C22—C21—H21B	109.5
C9—C8—H8A	108.1	H21A—C21—H21B	108.1
C7—C8—H8B	108.1	C23—C22—C21	112.7 (8)
C9—C8—H8B	108.1	C23—C22—H22A	109.1
H8A—C8—H8B	107.3	C21—C22—H22A	109.1
C10—C9—C8	110.9 (6)	C23—C22—H22B	109.1
C10—C9—H9A	109.5	C21—C22—H22B	109.1
C8—C9—H9A	109.5	H22A—C22—H22B	107.8
C10—C9—H9B	109.5	C22—C23—C24	111.3 (7)
C8—C9—H9B	109.5	C22—C23—H23A	109.4
H9A—C9—H9B	108.0	C24—C23—H23A	109.4
C11—C10—C9	114.2 (7)	C22—C23—H23B	109.4
C11—C10—H10A	108.7	C24—C23—H23B	109.4
C9—C10—H10A	108.7	H23A—C23—H23B	108.0
C11—C10—H10B	108.7	N1—C24—C23	108.3 (5)
C9—C10—H10B	108.7	N1—C24—H24A	110.0
H10A—C10—H10B	107.6	C23—C24—H24A	110.0
C10—C11—C12	114.4 (7)	N1—C24—H24B	110.0
C10—C11—H11A	108.7	C23—C24—H24B	110.0
C12—C11—H11A	108.7	H24A—C24—H24B	108.4
			
C7—Sn1—S1—C19	−74.8 (4)	C9—C10—C11—C12	51.7 (14)
C1—Sn1—S1—C19	62.3 (3)	C8—C7—C12—C11	36.6 (13)
C13—Sn1—S1—C19	176.1 (2)	Sn1—C7—C12—C11	−174.2 (6)
S2—Sn1—S1—C19	−4.49 (19)	C10—C11—C12—C7	−42.7 (12)
C7—Sn1—S2—C19	126.8 (3)	C7—Sn1—C13—C14	−176.0 (4)
C1—Sn1—S2—C19	−116.6 (2)	C1—Sn1—C13—C14	57.9 (5)
C13—Sn1—S2—C19	6.0 (4)	S1—Sn1—C13—C14	−57.9 (4)
S1—Sn1—S2—C19	4.69 (19)	S2—Sn1—C13—C14	−59.0 (6)
C7—Sn1—C1—C2	−75.0 (5)	C7—Sn1—C13—C18	−48.1 (5)
C13—Sn1—C1—C2	45.3 (5)	C1—Sn1—C13—C18	−174.2 (4)
S1—Sn1—C1—C2	147.8 (4)	S1—Sn1—C13—C18	70.0 (4)
S2—Sn1—C1—C2	−157.2 (4)	S2—Sn1—C13—C18	68.8 (6)
C7—Sn1—C1—C6	157.8 (5)	C18—C13—C14—C15	51.6 (7)
C13—Sn1—C1—C6	−81.9 (5)	Sn1—C13—C14—C15	179.7 (4)
S1—Sn1—C1—C6	20.6 (5)	C13—C14—C15—C16	−53.7 (8)
S2—Sn1—C1—C6	75.6 (4)	C14—C15—C16—C17	55.2 (8)
C6—C1—C2—C3	−53.1 (7)	C15—C16—C17—C18	−54.7 (8)
Sn1—C1—C2—C3	178.1 (5)	C14—C13—C18—C17	−52.1 (7)
C1—C2—C3—C4	55.0 (9)	Sn1—C13—C18—C17	178.4 (4)
C2—C3—C4—C5	−55.1 (9)	C16—C17—C18—C13	53.9 (8)
C3—C4—C5—C6	54.0 (9)	C20—N1—C19—S2	−171.2 (5)
C2—C1—C6—C5	51.1 (8)	C24—N1—C19—S2	−8.1 (8)
Sn1—C1—C6—C5	177.5 (5)	C20—N1—C19—S1	9.9 (8)
C4—C5—C6—C1	−51.9 (9)	C24—N1—C19—S1	173.1 (4)
C1—Sn1—C7—C8	−1.1 (9)	Sn1—S2—C19—N1	174.5 (5)
C13—Sn1—C7—C8	−123.9 (8)	Sn1—S2—C19—S1	−6.7 (3)
S1—Sn1—C7—C8	134.8 (8)	Sn1—S1—C19—N1	−172.4 (4)
S2—Sn1—C7—C8	78.4 (9)	Sn1—S1—C19—S2	8.6 (4)
C1—Sn1—C7—C12	−150.1 (6)	C19—N1—C20—C21	101.8 (8)
C13—Sn1—C7—C12	87.1 (7)	C24—N1—C20—C21	−62.8 (7)
S1—Sn1—C7—C12	−14.3 (8)	N1—C20—C21—C22	54.6 (9)
S2—Sn1—C7—C12	−70.7 (7)	C20—C21—C22—C23	−50.4 (10)
C12—C7—C8—C9	−39.0 (14)	C21—C22—C23—C24	51.7 (10)
Sn1—C7—C8—C9	172.4 (7)	C19—N1—C24—C23	−102.5 (7)
C7—C8—C9—C10	44.6 (14)	C20—N1—C24—C23	62.7 (7)
C8—C9—C10—C11	−51.1 (13)	C22—C23—C24—N1	−56.4 (9)
